# Circular RNA circ-CARD8 regulates alveolar macrophage pyroptosis through the miR-580-3p/CARD8 pathway in acute lung injury

**DOI:** 10.1371/journal.pone.0314936

**Published:** 2024-12-20

**Authors:** Sida Chen, Ling Wen, Yumei Wu, Shan Xiao, Yuting Lai, Jintao Ou, Yan Shen

**Affiliations:** 1 Respiratory Department, Longgang Central Hospital, Shenzhen, China; 2 Yadi Sancun Community Health Service Center, Shenzhen Pingle Orthopaedic Hospital (Shenzhen Pingshan Traditional Chinese Medicine Hospital), Shenzhen, China; Brigham and Women’s Hospital, UNITED STATES OF AMERICA

## Abstract

Pyroptosis is linked to the development of acute lung injury (ALI), and circular RNAs (circRNAs) play a role in ALI-related inflammation. However, the mechanisms by which circRNAs contribute to macrophage pyroptosis in ALI remain unclear. This study constructed an *in vitro* ALI model by inducing THP-1 cells with phorbol 12-myristate 13-acetate (PMA) and lipopolysaccharide (LPS). The expression and potential mechanism of circ-CARD8 in macrophage pyroptosis were then investigated. The interaction between circ-CARD8, hsa-miR-580-3p, and caspase recruitment domain family member 8 (CARD8) was confirmed through luciferase reporter assays and RNA-binding protein immunoprecipitation. Our data showed that circ-CARD8 was expressed at low levels. Meanwhile, the pyroptotic proteins caspase-1 and GSDMD, along with the secretion of chemokine (C-C motif) ligand 18 and interleukin 1 beta, were upregulated in the ALI cell model. Overexpression of circ-CARD8 reversed macrophage pyroptosis, whereas inhibition of circ-CARD8 promoted it. Furthermore, the expression of miR-580-3p, a downstream microRNA that binds to circ-CARD8, was reduced upon circ-CARD8 overexpression and increased following its inhibition. Additionally, overexpression of miR-580-3p suppressed the expression of CARD8, a downstream target of miR-580-3p, thereby promoting macrophage pyroptosis. The inhibition of miR-580-3p reversed the effect of circ-CARD8 silencing on macrophage pyroptosis and CARD8 expression. Therefore, our study confirms that the low expression of circ-CARD8 reduces the sponge adsorption of miR-580-3p, increasing its expression, which in turn targets and inhibits CARD8, ultimately promoting macrophage pyroptosis induced by LPS in THP-1 cells.

## Introduction

Acute lung injury (ALI) is a severe, life-threatening condition commonly caused by infection, trauma, or other critical health events, often leading to acute respiratory failure and multi-organ failure [1]. Despite advances in medical research and improved ventilation strategies, the mortality rate of sepsis-induced ALI remains as high as 35.5% [2]. The pathophysiology of ALI is complex and involves inflammatory cell migration, activation of apoptosis, increased membrane permeability, and dysregulation of coagulation, all of which impair alveolar gas exchange [3]. Macrophages are central to this process, playing critical roles in both the inflammatory response and the repair of damaged lung tissue, thus influencing the progression and outcome of ALI [4, 5].

Alveolar macrophages (AMs), the primary immune cells in the lungs, are essential for maintaining respiratory homeostasis and clearing pathogens and debris from the airways [4]. However, AMs can become overactivated during ALI, releasing pro-inflammatory cytokines and mediators that exacerbate lung damage [6, 7]. Besides to releasing cytokines, AMs can undergo pyroptosis, a unique form of inflammatory cell death that further drives the inflammatory environment in ALI [8, 9]. Pyroptosis is driven by the cleavage of gasdermin D (GSDMD) by caspase-1, resulting in cell lysis and the release of pro-inflammatory cytokines such as interleukin-1 beta (IL-1β) and interleukin-18 (IL-18) [10, 11]. This pyroptotic pathway is regulated by several factors, including caspase recruitment domain family member 8 (CARD8), a protein known to suppress inflammasome activation, and consequently, IL-1β release, thereby acting as a negative regulator of pyroptosis [12, 13]. Excessive macrophage pyroptosis has been observed in lipopolysaccharide (LPS)-induced ALI models, linking pyroptosis with lung inflammation and injury [14–16]. This connection between macrophage pyroptosis and lung inflammation suggests that targeting regulatory pathways in macrophage pyroptosis may offer potential therapeutic strategies for ALI.

One such regulatory pathway involves circular RNAs (circRNAs), a class of stable, non-coding RNAs with tissue-specific expression patterns recently emerging as critical regulators of immune responses and inflammation in various diseases, including ALI [17, 18]. CircRNAs are characterised by their covalently closed loop structure [19, 20]. And circRNAs were resistant to degradation and can function as "sponges" for microRNAs (miRNAs), competitively binding and sequestering miRNAs, thus modulating the expression of target genes [21, 22]. CircRNAs have been shown to regulate inflammation in ALI, further highlighting their role in lung injury. For example, circ0054633 is upregulated in LPS-induced lung injury, and its silencing reduces inflammation in lung endothelial cells [23]. Another circRNA, circRNA VAPA, inhibits macrophage pyroptosis through the miR-212-3p/Sirt1/Nrf2/NLRP3 axis, demonstrating the potential of circRNAs to modulate inflammatory cell death pathways relevant to ALI [24]. circN4bp1 promotes macrophage polarization and aggravates inflammation and lung injury in ALI mice by sponging micoRNA-138-5p [25]. These findings underscore the potential of circRNAs to influence macrophage functions in ALI, suggesting they may serve as valuable therapeutic targets in inflammatory lung diseases.

Circ-CARD8 (hsa_circ_0002749) is particularly interesting because it originates from the CARD8 gene, a known negative regulator of inflammasome activation [26]. CARD8, a component of the inflammasome complex, is known to inhibit inflammasome activation and thereby limit IL-1β release and pyroptosis [27, 28]. Since immune dysregulation and excessive inflammation are critical drivers in ALI [29], we hypothesized that circ-CARD8 might play a regulatory role in modulating macrophage pyroptosis, thus influencing the inflammatory landscape in ALI. However, despite its potential importance, the functional role of circ-CARD8 in ALI, particularly in the context of macrophage pyroptosis, remains unexplored.

In our study, we investigated the role of circ-CARD8 in ALI by constructing an *in vitro* ALI model using THP-1 cells stimulated with phorbol 12-myristate 13-acetate (PMA) and LPS. In this model, we examined the expression and regulatory mechanism of circ-CARD8. We identified its downstream miRNA targets by intersecting the predicted target miRNAs of circ-CARD8 (using circAtlas 2.0) with those regulating CARD8 expression (using TargetScanHuman 7.1). Among the overlapping miRNAs, we selected miR-580-3p, which has dual binding sites with both circ-CARD8 and CARD8, for further analysis. Our results revealed that circ-CARD8 expression was downregulated during LPS-induced macrophage pyroptosis, leading to reduced miR-580-3p sponging, an increase in miR-580-3p expression, and subsequent inhibition of CARD8, which promoted macrophage pyroptosis. This suggests that circ-CARD8 downregulation may exacerbate inflammatory responses in ALI by enhancing macrophage pyroptosis. Although our study primarily provides *in vitro* data, we further investigated the clinical relevance of circ-CARD8 by examining its expression in peripheral blood mononuclear cells (PBMCs) from sepsis-induced ALI patients.

Therefore, our findings suggest that circ-CARD8 may serve as a biomarker for ALI progression and severity and offers promise as a potential therapeutic target to modulate inflammation in ALI.

## Methods and materials

### Patients and peripheral blood mononuclear cells (PBMCs) isolation

Blood samples were collected from 20 cases of patients with sepsis-induced ALI at Longgang Central Hospital, as well as 20 cases of control peripheral blood samples. The inclusion criteria for patients with sepsis-induced ALI were as follows: 1) diagnosed with sepsis; 2) SOFA score ≥ 2; 3) presence of an infectious focus accompanied by an inflammatory response. The control group consists of healthy volunteers. Exclusion criteria included: 1) age < 18 or > 80 years; 2) pregnancy; 3) hematological diseases, cancer, or autoimmune diseases; 4) chemotherapy or hormone therapy within the last 4 weeks. All procedures of this study were approved by the Ethics Committee of Longgang Central Hospital (Approval No.: 2024ECPJ086) and were in accordance with the principles of the Declaration of Helsinki, and all participants signed written informed consent. PBMCs were isolated from blood samples using human whole blood mononuclear cell separation liquid (LDS1075, TBD, Tianjin, China), followed by RNA extraction to detect the expression of circ-CARD8 in PBMCs.

### Cell culture

THP-1 cells were obtained from Cellcook (CC1904, Guangzhou, China) and cultured in RPMI 1640 medium (11875, GIBCO) supplemented with 10% fetal bovine serum and 0.05 mM mercaptoethanol. The cells were incubated with 100 ng/mL PMA (HY-18739, MCE) for 48 h to facilitate adhesion. Subsequently, different concentrations of LPS (HY-D1056, MCE) (0, 0.1, 0.3, 1, 3 μg/mL) were added for 48 h of induction, or a concentration of 1 μg/mL LPS was applied for varying durations (0, 12, 24, 48 h). Afterwards, cells were collected for subsequent experiments.

### Cell transfection

Plasmids overexpressing circ-CARD8, small interfering RNA (siRNA) fragments of circ-CARD8, miR-580-3p mimics, and miR-580-3p inhibitor were synthesized by Jiangsu Saisuofei Biology. THP-1 cells were seeded into 24-well plates, incubated with 100 ng/mL PMA for 48 h, and the medium was aspirated after the cells adhered. Dissolve 5 μL of the siRNA fragment, 0.5 μg plasmid and 0.5 μg of the internal control plasmid in 250 μL of opti-MEM. Lipofectamine 2000 (BL623B, Biosharp) was used for transfection, and after 4 h, a fresh complete medium was replaced, and the culture was continued until 24 h.

The si-circ-CARD8-1 sequences: 5’-CTCCTCCTTTCTCAGGCGATA-3’,

The si-circ-CARD8-2 sequences: 5’-CTCCTTTCTCAGGCGATAGAT-3’,

The si-circ-CARD8-3 sequences: 5’-TTCTCAGGCGATAGATGATGA-3’,

The si-NC sequences: 5’-CATGCCGACTCCTGGGGCAGG-3’.

### Cell activity

Cell Counting Kit-8 reagent (CCK-8, G4103, Servicebio) was used to detect cell activity after indicated treatment, like the previous study, with a few revisions [30]. In brief, 3000 cells were inoculated into 96-well plates, and 10 μL CCK-8 was added into each well after the indicated treatment. The absorbance of 450 was detected after 2 h.

### Fluorescence in situ hybridization (FISH)

The FISH experiment was performed following the previous study [30]. The probe sequence of circ-CARD8: CATCTATCGCCTGAGAAAGG-CY5. Leica DM5500B microscope (Leica, Germany) was used to acquire images.

### Western blot

Western blot was performed following the previous study [30]. The primary antibody was as follows: anti-Caspase-1 (ab286125, Abcam), anti-GSDMD (ab219800, Abcam), anti-cleaved N-terminal GSDMD (ab215203, Abcam), anti-CARD8 (ab24186, Abcam) and anti-β-actin (20536-1-AP, Proteintech). The secondary antibody was horseradish peroxidase-Goat Anti-Rabbit IgG (H+L) (AS014, AB clonal).

### Quantitative real-time polymerase chain reaction (qRT-PCR)

Total cell RNA was extracted using the TriQuick Reagent total RNA extraction reagent (Solarbio, Beijing, China). Following RNA extraction, HiScript III RT SuperMix for qPCR (R232, Vazyme) was used for reverse transcription to produce complementary DNA, and the relative expression of the target gene was detected by qPCR using ChamQ Universal SYBR qPCR Master Mix (Q711, Vazyme). The quantity calculation formula is 2^-ΔΔCt^ = 2^-【(ΔCt)Test—(ΔCt)Control】^. The primer sequences are listed in Table 1. The cycling condition was set as follows: 95°C, 30 sec, one cycle; 95°C 10 sec, 60°C, 30 sec, 40 cycles.

**Table 1:** The primers used in this study.

Names	Sequences (5’to3’)
circ-CARD8-F	GCTGGGCAGATGAAGGAAC
circ-CARD8-R	TCTTCCTCATCATCTATCGCCTG
CARD8-F	CCGAGACGGGTATACAGGGA
CARD8-R	GGCTCTGCCTCTGTCTCATC
H-GAPDH F	GAGTCAACGGATTTGGTCGT
H-GAPDH R	GACAAGCTTCCCGTTCTCAG
hsa-miR-580-3p-RT	GTCGTATCCAGTGCAGGGTCCGAGGTATTCGCACTGGATACGACAACTCT
hsa-miR-580-3p-F	TTGAGAATGATGAATCATTAGG
U6-F	CTCGCTTCGGCAGCACA
U6-R	AACGCTTCACGAATTTGCGT

### Analysis of chemokine (C-C motif) ligand 18 (CCL18) and IL-1β

Human lung activation modulating chemokine (PARC/CCL18) ELISA kits (CUSABIO, CSB-E09941h) and human interleukin-1β (IL-1β) enzyme-linked immunosorbent assay (ELISA) kits (CUSABIO, CSB-E08053h) were used to detect the content of CCL18 and IL-1β in THP-1 cell supernatant base on the instruction of kit.

### Dual-luciferase reporter gene assay

Like the previous study [31], the TransDetect Double-Luciferase Reporter Assay Kit (Transgen, FR201-01) was used to detect luciferase activity.

### Argonaute 2-RIP: RNA-binding protein immunoprecipitation (AGO2-RIP) assay

Following a previous study, RIP experiments were performed using the Magna RIP^™^ RNA-Binding Protein Immunoprecipitation Kit (17–700, Millipore) [30]. The collected RNA was reverse transcribed (HiScript III RT SuperMix for qPCR), followed by qPCR detection.

### Statistical analysis

All data are presented as mean ± standard deviation (SD), and three independent experiments were performed for each group of experiments. Statistical calculations were performed using GraphPad Prism 8.0 software, and the student t-test was used to compare the differences between the two groups. One-way ANOVA was used to evaluate the difference between three or more groups. *P* < 0.05 was considered statistically significant.

## Results

### Hsa_circ_0002749 originates from the CARD8 exon and was lowly expressed in the PBMC of ALI patients

**Fig 1:**
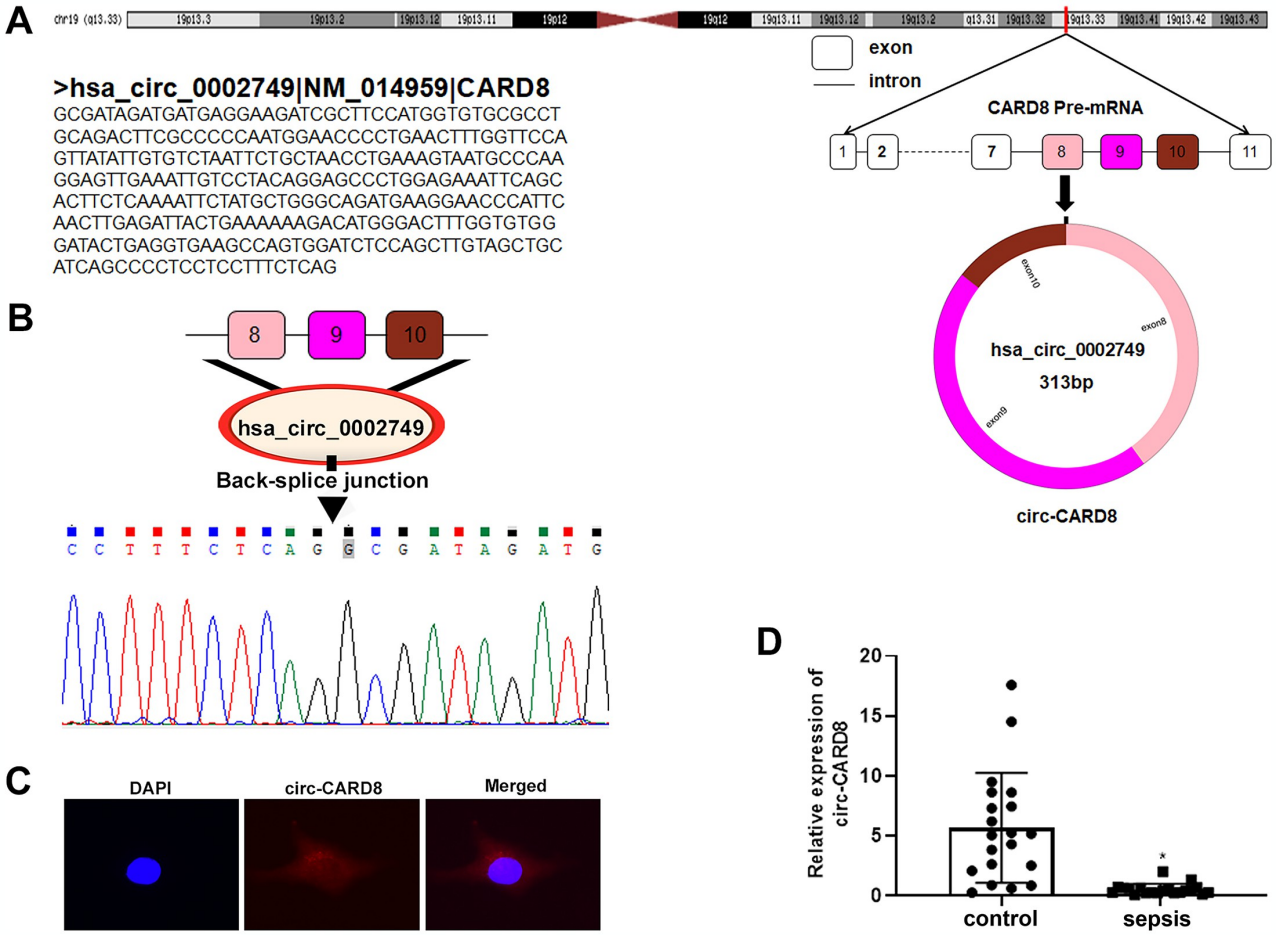
Identification and expression of hsa_circ_0002749 in patients with sepsis-induced ALI. (A) University of California Santa Cruz (UCSC) combined circPrimer to analyze the source of hsa_circ_0002749. (B) Sanger sequencing detected the presence of a circ-CARD8 circularization site. (C) Detection of subcellular localization of circ-CARD8 in macrophages by fluorescence in situ hybridization. (D) qRT-PCR examined the expression of circ-CARD8 in the PBMC of patients with sepsis-induced ALI and healthy individuals. *P < 0.05.

University of California Santa Cruz (UCSC) combined with circPrimer analysis revealed that hsa_circ_0002749 originated from exon 8–10 of CARD8, so it was labelled as circ-CARD8 (Fig 1A). Sanger sequencing confirmed the existence of circ-CARD8 cyclization site (Fig 1B). Then FISH was performed to detect the localization of circ-CARD8 in macrophages. It was observed that circ-CARD8 was mainly located in the cytoplasm (Fig 1C). qRT-PCR results displayed that circ-CARD8 expression was downregulated in PBMCs from patients with ALI relative to healthy controls, indicating a potential role of circ-CARD8 in the pathophysiology of ALI (Fig 1D).

### LPS inhibited the expression of circ-CARD8 and promoted pyroptosis of macrophage

**Fig 2:**
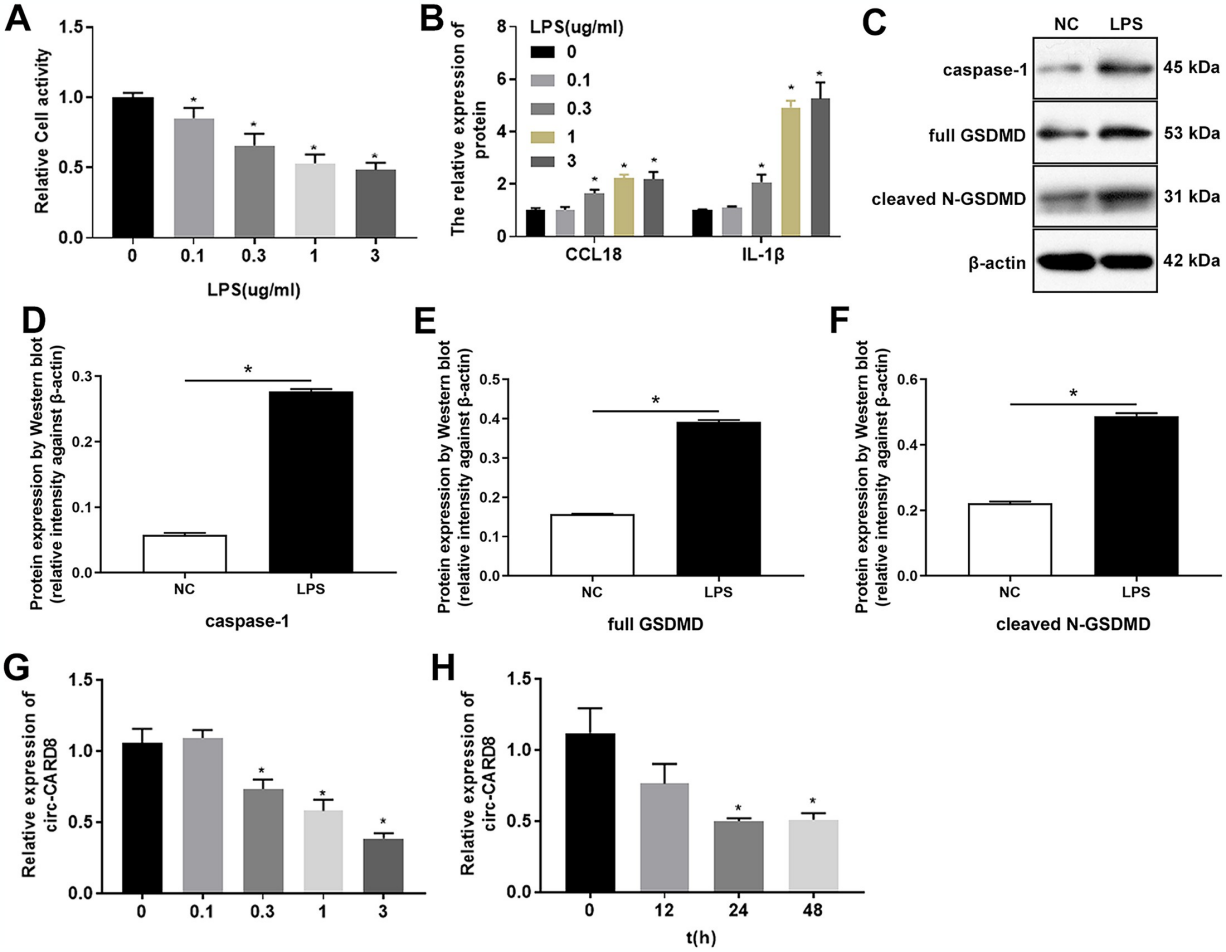
Lipopolysaccharide (LPS) inhibited the expression of circ-CARD8 and promoted pyroptosis of macrophage cells. THP-1 was incubated with 100 ng/mL PMA for 48 h to induce macrophage. (A) After being treated with various concentrations of LPS (0, 0.1, 0.3, 1, 3 μg/mL) for 48 h, CCK-8 was used to detect the activity of macrophage cells. (B) After being treated with different concentrations of LPS (0, 0.1, 0.3, 1, 3 μg/mL) for 48 h, the supernatant of macrophage cells was collected, and the contents of chemokine (C-C motif) ligand 18 (CCL18) and interleukin 1 beta (IL-1β) were examined by ELISA kit. (C-F) After being treated with 1 μg/mL LPS, the protein expressions of caspase-1 and GSDMD in macrophage cells were tested by western blot assay. (G) After treating with different LPS concentrations (0, 0.1, 0.3, 1, 3 μg/mL) for 48 h, the expression of circ-CARD8 was analyzed by quantitative real-time polymerase chain reaction. (H) LPS (1 μg/mL) treated macrophage for different time points (0, 12, 24, 48 h), and the expression of circ-CARD8 was detected by quantitative real-time polymerase chain reaction. *P < 0.05.

In the *in vitro* model of ALI induced by LPS, we observed that the cell activity of macrophage, PMA-induced THP-1 cells, decreased significantly with increasing LPS concentration (Fig 2A). The concentrations of CCL18 and IL-1β in the supernatant of LPS-treated macrophage cells increased in an LPS-dependent manner (Fig 2B). Since the experimental phenomena of 1 μg/mL and 3 μg/mL treatment were similar, 1 μg/mL LPS was selected as the concentration in subsequent studies. Besides, the results revealed that LPS promoted the expression of caspase-1 and GSDMD, the essential proteins of macrophage pyroptosis (Fig 2C–2F). We further investigated the effect of LPS on circ-CARD8, and the results showed that the expression of circ-CARD8 attenuated with the increase of LPS concentration and treatment time (Fig 2G and 2H).

### LPS regulated macrophage pyroptosis via targeting circ-CARD8

**Fig 3:**
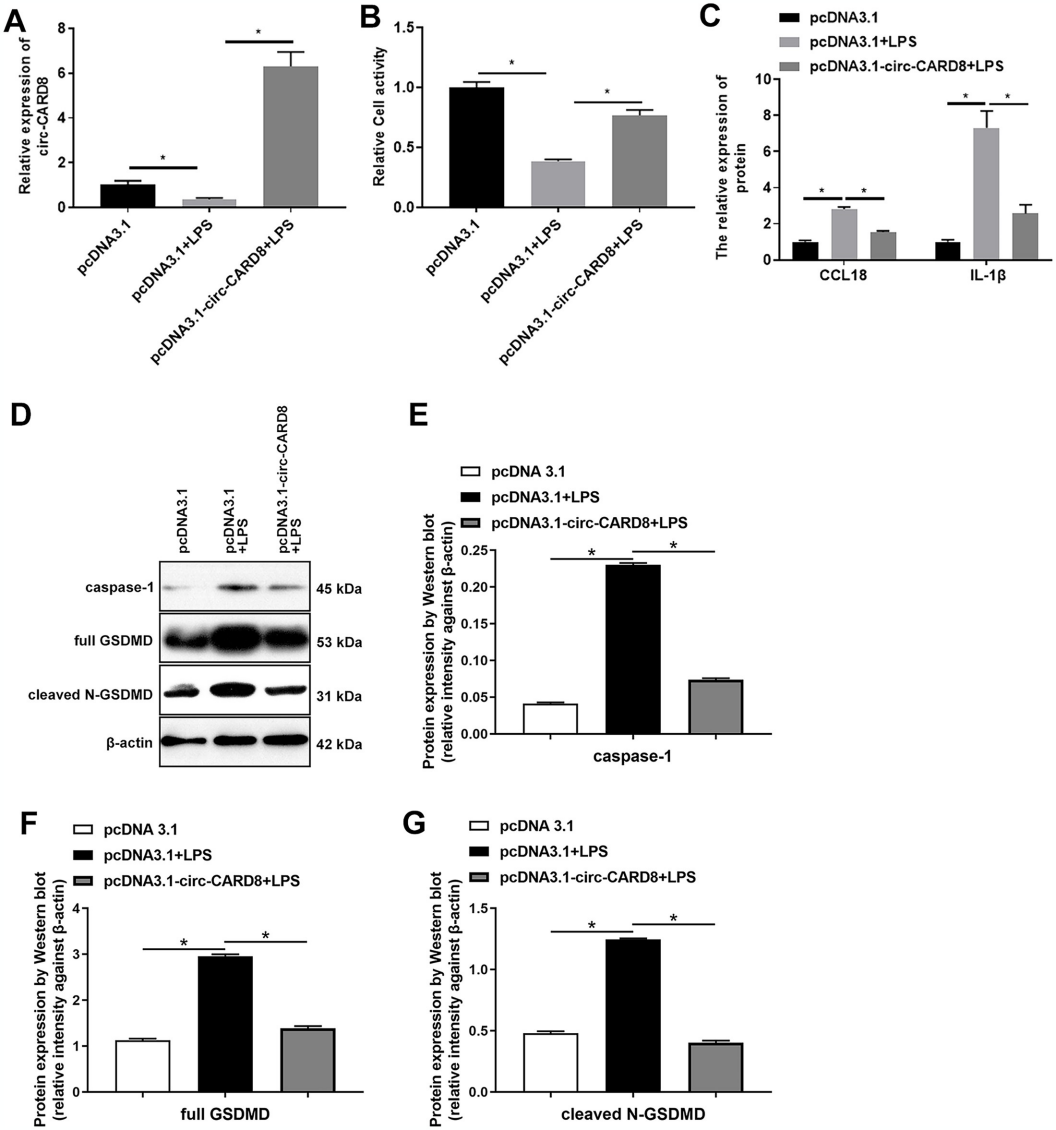
Overexpression of circ-CARD8 inhibited Lipopolysaccharide (LPS)-induced macrophage pyroptosis. THP-1 cells were incubated with 100 ng/mL PMA for 48 h to induce macrophage; then, the circ-CARD8 plasmid was transfected into a macrophage. After 12 h, 1 μg/mL LPS was added to the treatment for 48 h. (A) Quantitative real-time polymerase chain reaction detected the expression of circ-CARD8. (B) CCK-8 detected the cell activity. (C) ELISA evaluated the contents of chemokine (C-C motif) ligand 18 (CCL18) and interleukin 1 beta (IL-1β) in the supernatant. (D-G) Western blot assay detected the expression of pyroptosis protein caspase-1 and GSDMD. *P < 0.05.

To explore whether circ-CARD8 plays a role in LPS-induced macrophage pyroptosis, we constructed circ-CARD8 overexpression plasmid. Circ-CARD8 plasmid markedly upregulated the down-regulation of circ-CARD8 expression caused by LPS in macrophage, indicating that the pcDNA3.1-circ-CARD8 plasmid construction was successful (Fig 3A). By detecting macrophage cell activity, we found that overexpression of circ-CARD8 reversed the decline in cell activity induced by LPS in macrophage (Fig 3B). High levels of CCL18 and IL-1β induced by LPS in macrophage decreased after overexpression of circ-CARD8 (Fig 3C). The elevation of pyroptosis essential proteins caspase-1 and GSDMD also attenuated after overexpression of circ-CARD8 (Fig 3D–3G).

**Fig 4:**
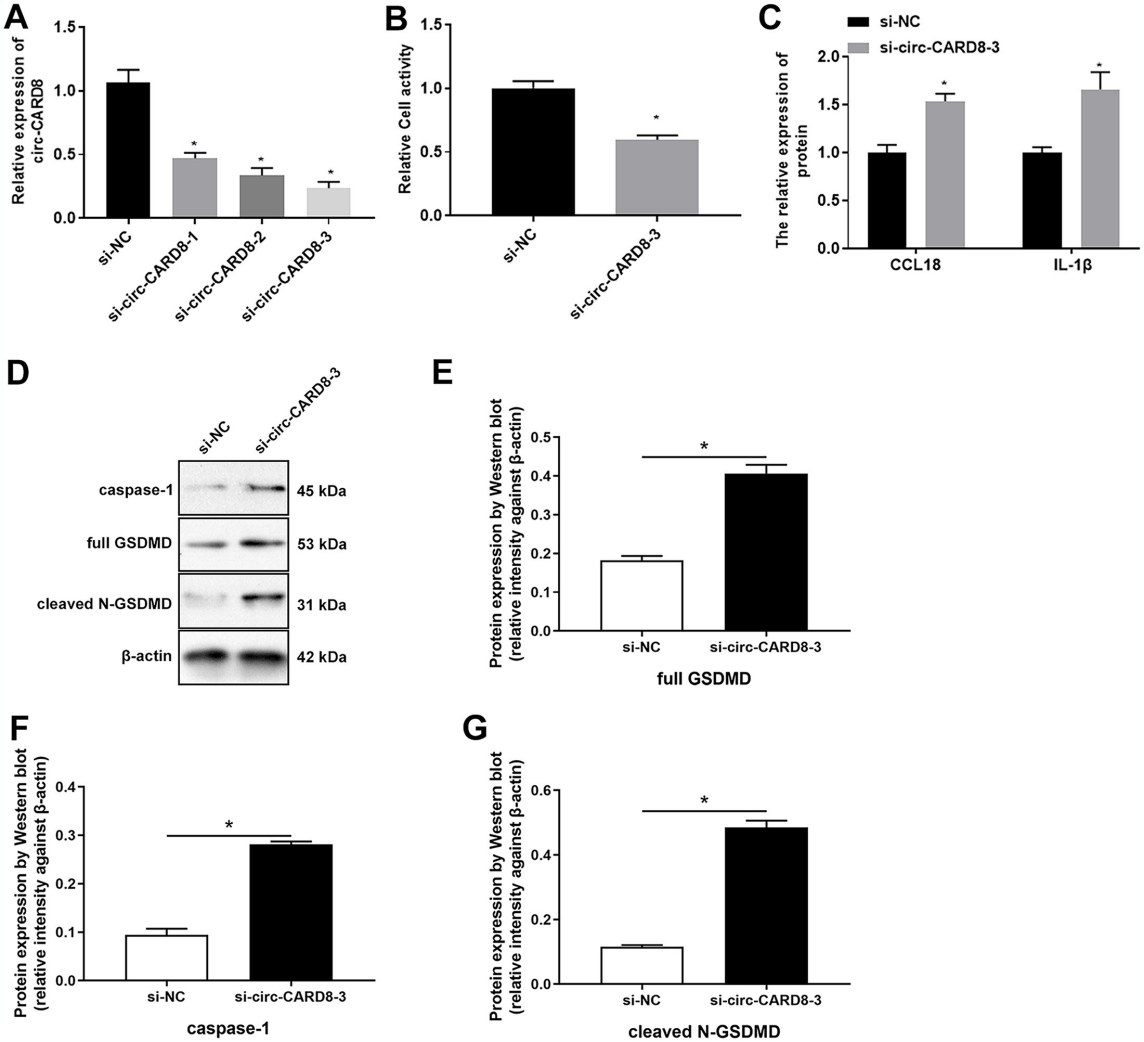
Inhibition of circ-CARD8 promoted pyroptosis of macrophages. THP-1 was incubated with 100 ng/mL PMA for 48 h to induce macrophage; then, the small interfering RNA fragments that interfered with circ-CARD8 (si-circ-CARD8) were transfected into macrophage. (A) Quantitative real-time polymerase chain reaction detected the expression of circ-CARD8. (B) CCK-8 detected the cell activity. (C) ELISA evaluated the contents of chemokine (C-C motif) ligand 18 (CCL18) and interleukin 1 beta (IL-1β) in the supernatant. (D-G) Western blot assay detected the expression of pyroptosis protein caspase-1 and GSDMD. *P < 0.05.

Besides, we utilized siRNA to inhibit the expression of circ-CARD8 in macrophages. The results showed that si-circ-CARD8-1/2/3 all decreased the circ-CAR8 expression, in which si-circ-CARD8-3 had the most obvious effect, which was used in subsequent experiments (Fig 4A). When the expression of circ-CARD8 was eliminated, we observed that the activity of macrophage cells decreased. The contents of CCL18 and IL-1β in cell supernatant increased (Fig 4B and 4C). Moreover, inhibition of circ-CARD8 expression resulted in the upregulation of pyroptosis proteins caspase-1 and GSDMD (Fig 4D–4G).

### Circ-CARD8 negatively regulated the expression of miR-580-3p

**Fig 5:**
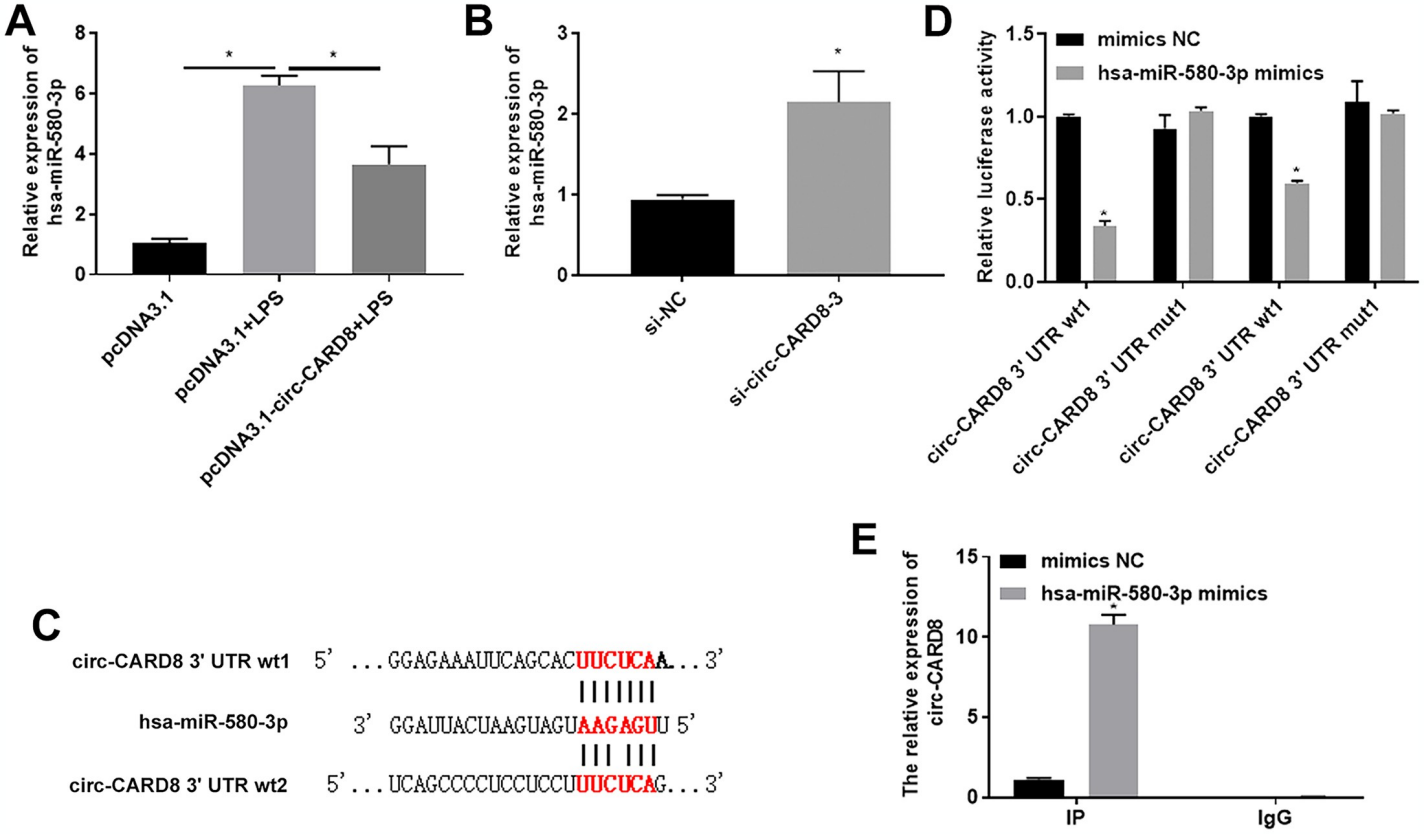
circ-CARD8 negatively regulated the expression of miR-580-3p. (A) After overexpression of circ-CARD8, the expression of miR-580-3p in LPS-treated macrophages was assessed by quantitative real-time polymerase chain reaction. (B) The expression of miR-580-3p after interference with circ-CARD8 was detected by quantitative real-time polymerase chain reaction. (C) The binding sequence between circ-CARD8 and miR-580-3p was predicted by circAtlas 2.0 software. (D) The binding site between circ-CARD8 and miR-580-3p was verified by dual-luciferase reporter gene assay. (E) The binding between miR-580-3p and circ-CARD8 was detected by argonaute 2-RNA-binding protein immunoprecipitation (AGO2-RIP) assay. *P < 0.05.

Then, we verified whether circ-CARD8 regulated miR-580-3p. Firstly, we detected the expression of miR-580-3p after changing the expression of circ-CARD8. The expression of miR-580-3p was distinctly increased under the treatment of LPS on the macrophage but could be reversed by overexpression of circ-CARD8 (Fig 5A). However, miR-580-3p expression was upregulated after transfected with si-circ-CARD8-3 in macrophage (Fig 5B). Then, we showed the binding sequence between circ-CARD8 and miR-580-3p predicted by circles 2.0 software. The binding site between them was confirmed using dual luciferase reporter gene detection (Fig 5C and 5D). Furthermore, the RIP experiment further confirmed the binding between miR-580-3p and circ-CARD8 (Fig 5E).

### miR-580-3p targeted CARD8 to promote macrophage pyroptosis

**Fig 6:**
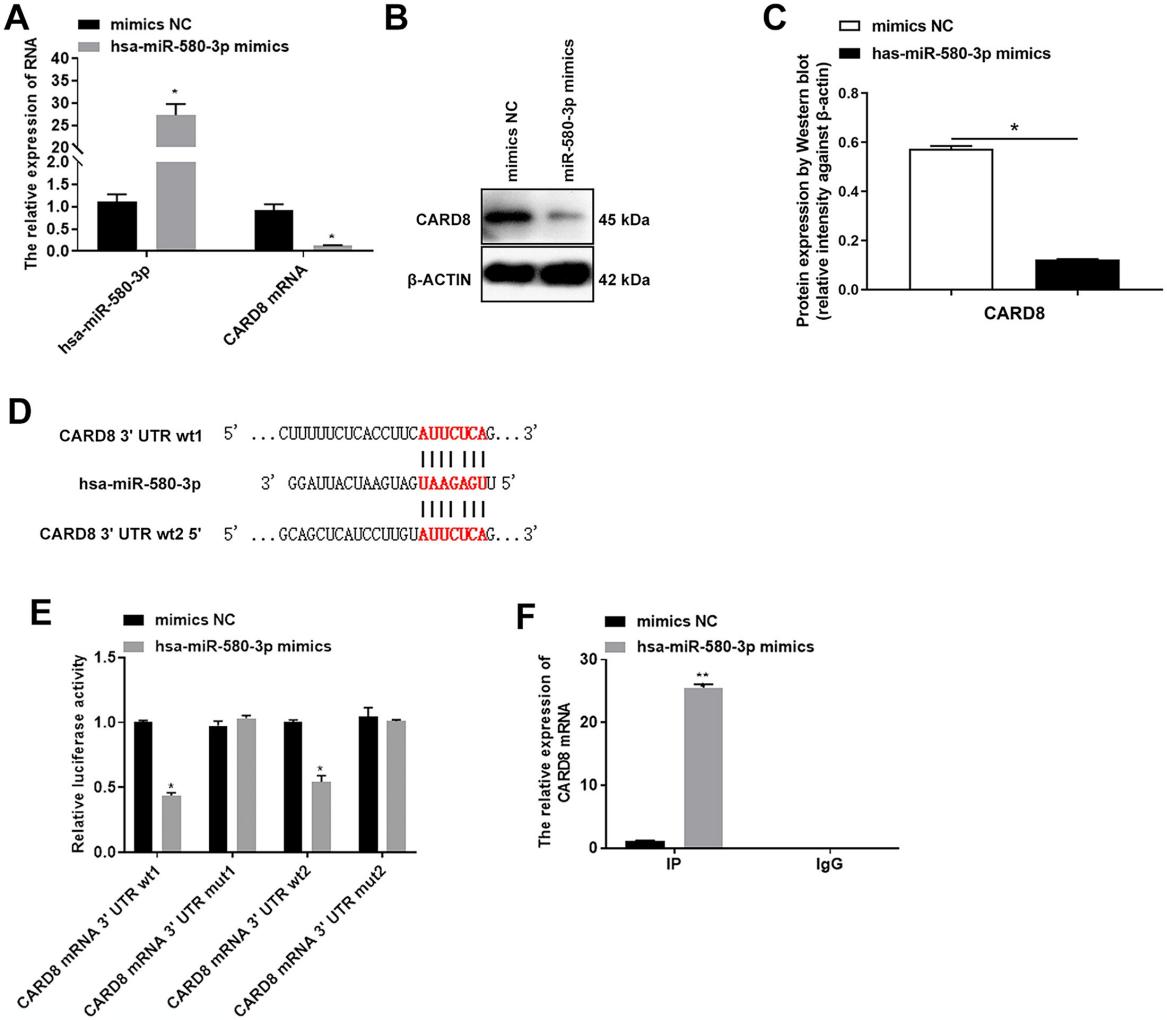
miR-580-3p inhibited the expression of caspase recruitment domain family member 8 (CARD8). (A) After overexpression of miR-580-3p, the expression of miR-580-3p and CARD8 was detected by quantitative real-time polymerase chain reaction. (B-C) After overexpression of miR-580-3p, the protein expression of CARD8 was detected by western blot assay. (D) TargetScanHuman predicted the binding site of miR-580-3p and CARD8 3’ UTR (untranslated regions) 7.1. (E) Detection of binding sites between CARD83’ UTR and miR-580-3p by dual-luciferase reporter gene assay. (F) Argonaute 2-RNA-binding protein immunoprecipitation (AGO2-RIP) assay detected the binding between miR-580-3p and CARD8. *P < 0.05.

As a pyroptosis suppressor gene, the decreased expression of CARD8 can facilitate the secretion of IL-1β [32]. To explore whether miR-580-3p targets CARD8 to participate in macrophage pyroptosis, we first examined the expression levels of CARD8 after overexpression of miR-580-3p. It was demonstrated that overexpression of miR-580-3p inhibited the expression of CARD8 (Fig 6A–6C). Then the binding site of miR-580-3p to the 3’ UTR (untranslated regions) of CARD8 was predicted by TargetScanHuman 7.1 software (Fig 6D). And the results of dual-luciferase reporter gene assay indicated that there was a binding site between CARD83’ UTR and miR-580-3p (Fig 6E). RIP experiments further confirmed the binding of miR-580-3p to CARD8 (Fig 6F).

**Fig 7:**
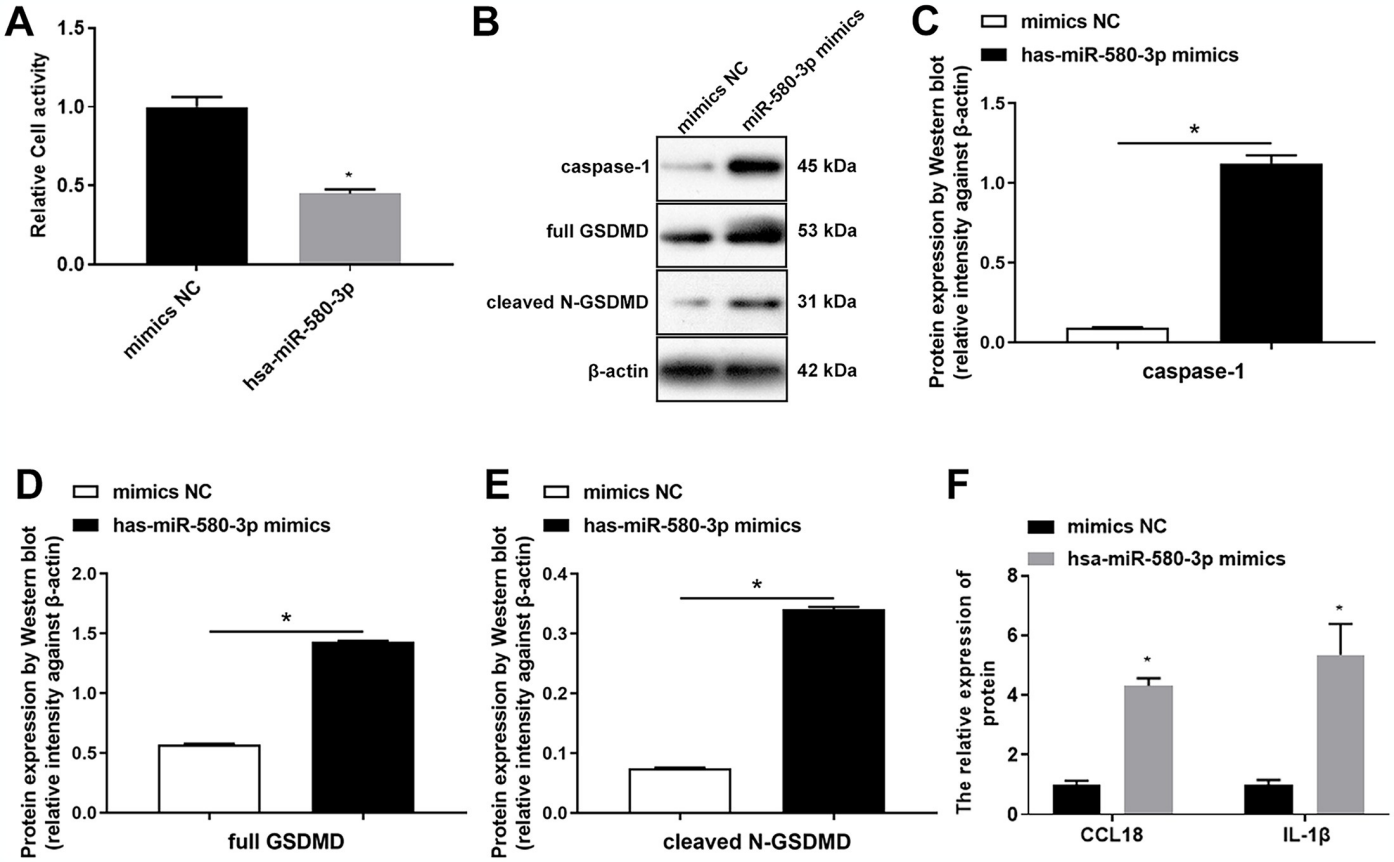
Overexpression of miR-580-3p promoted pyroptosis of macrophages. THP-1 was incubated with 100 ng/mL PMA for 48 h, and after cells adhered to induce macrophage, then miR-580-3p mimics were transfected into macrophage for 48 h. (A) CCK-8 detected the cell activity. (B-E) Western blot assay detected the expression of pyroptosis protein caspase-1 and gasdermin D (GSDMD). (F) ELISA evaluated the contents of chemokine (C-C motif) ligand 18 (CCL18) and interleukin 1 beta (IL-1β) in the supernatant. *P < 0.05.

Furthermore, we found that overexpression of miR-580-3p inhibited macrophage cell activity (Fig 7A). Overexpression of miR-580-3p promoted the expression of caspase-1 and GSDMD (Fig 7B–7E). Moreover, the contents of CCL18 and IL-1β in cell supernatants were upregulated by overexpression of miR-580-3p (Fig 7F).

### Circ-CARD8 promoted macrophage pyroptosis by regulating the expression of CARD8 through targeting miR-580-3p

**Fig 8:**
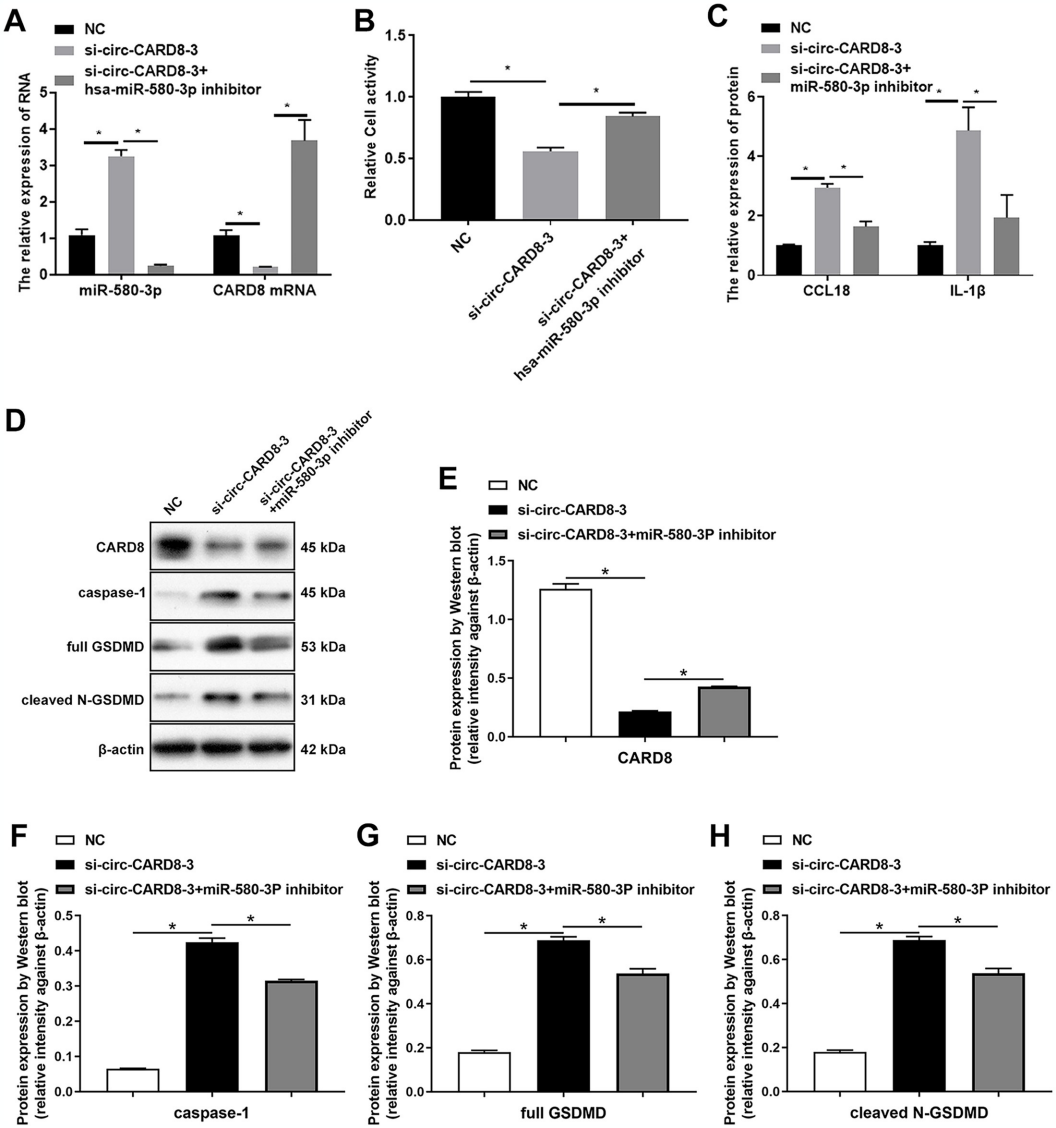
circ-CARD8 promoted macrophage pyroptosis by regulating miR-580-3p/caspase recruitment domain family member 8 (CARD8). THP-1 was incubated with 100 ng/mL PMA for 48 h to induce macrophage, and then si-circ-CARD8-3 and miR-580-3p inhibitor were transfected into macrophage. (A) Quantitative real-time polymerase chain reaction was used to evaluate the expression of miR-580-3p and CARD8. (B) CCK-8 detected the cell activity. (C) ELISA evaluated the contents of chemokine (C-C motif) ligand 18 (CCL18) and interleukin 1 beta (IL-1β) in the supernatant. (D-H) Western blot assay detected the expression of pyroptosis protein caspase-1 and gasdermin D (GSDMD). *P < 0.05.

To confirm that circ-CARD8 promoted macrophage pyroptosis by regulating CARD8 expression through targeting miR-580-3p, we transfected with si-circ-CARD8-3 and miR-580-3p inhibitor into macrophage. Results showed that the expression of CARD8 decreased after inhibition of circ-CARD8. In contrast, the expression of CARD8 was restored after inhibiting miR-580-3p (Fig 8A). Inhibition of miR-580-3p significantly restored the impaired cell activity and reduced the elevation of CCL18 and IL1β caused by si-circ-CARD8-3 (Fig 8B and 8C). The increased protein levels of caspase-1 and GSDMD induced by inhibition of circ-CARD8 also decreased after inhibiting miR-580-3p (Fig 8D–8H).

## Discussion

ALI is an intractable disease worldwide with complex pathogenesis [33]. More and more researchers have validated that circRNAs regulate essential proteins of cellular inflammatory responses, such as alveolar macrophages in ALI inflammatory responses, play a regulatory role through various signaling pathways, and may be a therapeutic target for ALI [34, 35]. Only one previous study found circRNA VAPA involved in macrophage pyroptosis during ALI [24]. However, there is still a lack of information on the effect of other circRNAs on macrophage pyroptosis during ALI progression. In the study of human ALI, LPS-induced experimental models have been widely used [36]. In the present study, we established an LPS-induced ALI model *in vitro* to explore the possible mechanism by which circ-CARD8 induces macrophage pyroptosis in ALI.

Pyroptosis is an inflammation-induced programmed cell death [37]. The classical pyroptotic pathway generally manifests as an inflammasome complex containing a precursor of caspase-1, which is then cleaved to generate active caspase-1, ultimately leading to the cleavage of the substrate GSDMD. The N-terminus of the cleaved GSDMD forms the hole in the host cell membrane, which releases various inflammatory factors, including IL-1β and CCL18, causing inflammation and pyroptosis [38]. In the LPS-induced ALI model, LPS activated alveolar macrophage pyroptosis by regulating the NLR family pyrin domain containing 3/ASC/caspase-1 inflammasome complex through the p38 MAPK pathway [39, 40]. In this experiment, the N-terminal expression level of GSDMD was potently increased, and the levels of caspase-1 were also increased. The levels of IL-1β and CCL18 were substantially upregulated, suggesting that LPS promotes pyroptosis in macrophages.

Circular RNAs (circRNAs) commonly regulate target gene expression by sponging miRNAs, thereby influencing downstream signaling pathways [41]. In ALI, specific circRNAs could modulate lung inflammation by interacting with various miRNAs. For example, circ0001434 was downregulated in LPS-induced ALI, and its overexpression targeted the nuclear factor kappa B signaling pathway by sponging miR-625-5p, which alleviated lung inflammation [42]. Similarly, circ0054633 was highly expressed in LPS-induced ALI, and its knockdown markedly reduced lung injury [23]. Our study further supported the role of circRNAs in ALI by demonstrating that circ-CARD8 was significantly downregulated in LPS-induced ALI. Overexpression of circ-CARD8 led to increased macrophage viability, reduced caspase-1 and gasdermin D (GSDMD) expression, and decreased secretion of pro-inflammatory mediators such as CCL18 and IL-1β, collectively reducing LPS-induced macrophage pyroptosis. Given that CARD8 is a known negative regulator of pyroptosis, our findings suggested that circ-CARD8 modulated macrophage inflammatory responses and cell death by influencing CARD8 expression. CARD8 knockdown has been shown to activate caspase-1 and trigger the release of large amounts of IL-1β, further supporting its role as a pyroptosis suppressor [32, 43].

Our bioinformatics analysis also identified miR-580-3p as a central component of this regulatory axis, as it possessed multiple binding sites with circ-CARD8 and CARD8. Although miR-580-3p had mainly been studied in cancer research, which influenced cell proliferation, metastasis, and autophagy [44–46], its potential role in ALI had not been previously investigated. We hypothesized that miR-580-3p might mediate the effects of circ-CARD8 on CARD8 expression in macrophages during ALI. Our study observed that miR-580-3p expression increased significantly in LPS-induced ALI but was suppressed when circ-CARD8 was overexpressed. This interaction between circ-CARD8 and miR-580-3p suggested that circ-CARD8 likely acted as a molecular sponge, inhibiting miR-580-3p and thereby preserving CARD8 function in preventing pyroptosis.

Furthermore, we demonstrated that overexpression of miR-580-3p inhibited CARD8 expression, promoted caspase-1 and GSDMD activity, and enhanced macrophage secretion of CCL18 and IL-1β, indicating its pro-pyroptotic effects. This implied that miR-580-3p was pivotal in regulating macrophage pyroptosis and inflammatory responses in ALI by targeting CARD8. This finding expanded our understanding of miRNA involvement in inflammatory lung injury. Therefore, circ-CARD8’s ability to sequester miR-580-3p added a vital layer to its regulatory effects in ALI, suggesting its potential as a therapeutic target for controlling inflammation and pyroptosis in macrophages.

## Conclusions

This investigation proposes a potential mechanism whereby circ-CARD8 regulates the expression of caspase-1 and GSDMD by sponging miR-580-3p targeting CARD8 to promote macrophage pyroptosis in LPS-induced ALI. Besides, the low expression of circ-CARD8 may become a clinical monitoring indicator for severe ALI, and it is expected to become a therapeutic target for ALI.
